# Performance of international prognostic indices in plasmablastic lymphoma: a comparative evaluation

**DOI:** 10.1007/s00432-021-03580-z

**Published:** 2021-03-03

**Authors:** Nadine Hertel, Hartmut Merz, Heinz-Wolfram Bernd, Veronica Bernard, Axel Künstner, Hauke Busch, Nikolas von Bubnoff, Alfred C. Feller, Hanno M. Witte, Niklas Gebauer

**Affiliations:** 1 Department of Hematology and Oncology, University Hospital of Schleswig-Holstein, Campus Lübeck, Ratzeburger Allee 160, 23538 Lübeck, Germany; 2 Reference Centre for Lymph Node Pathology and Hematopathology, Hämatopathologie Lübeck, Lübeck, Germany; 3 Medical Systems Biology Group, University of Lübeck, Ratzeburger Allee 160, 23538 Lübeck, Germany; 4 Institute for Cardiogenetics, University of Lübeck, Ratzeburger Allee 160, 23538 Lübeck, Germany; 5 Department of Hematology and Oncology, Federal Armed Forces Hospital Ulm, Oberer Eselsberg 40, 89081 Ulm, Germany

**Keywords:** Plasmablastic lymphoma, IPI, Prognosis, Risk stratification

## Abstract

**Purpose:**

Plasmablastic lymphoma (PBL) is a rare and aggressive B-cell malignancy with a heterogenous clinical and prognostic spectrum, determined by multiple factors, including age, HIV- and *MYC*-status. While there exist several validated scoring systems for diffuse large B-cell lymphoma, which incorporate basic clinical features (age, lactate dehydrogenase, sites of (extranodal) involvement, stage and performance), none of these have been systematically assessed in PBL.

**Methods:**

We determined the (age-adjusted; aa)-International Prognostic Index (IPI), revised IPI (R-IPI), and National Comprehensive Cancer Network IPI (NCCN-IPI) in a comprehensive multi-center cohort (*n* = 78) of PBL patients. Further, all indices were comparatively investigated for model quality and concordance.

**Results:**

Univariate analysis revealed significant prognostic capabilities for all indices, all of which identified a subgroup with favorable outcome. Discriminatory power between patients with less benign prognosis and especially refractory disease exhibited significant variability. Subsequently, stratified models for each risk score were compared employing corrected Akaike’s information criterion (cAIC) and Harrel’s concordance index (c-index). Here, the NCCN-IPI outperformed both IPI and R-IPI regarding c-index with ambiguous cAIC results, underlining its clinical utility and suggesting it for preferential use in clinical practice.

**Conclusion:**

Our current observations support the use of the IPI and its enhanced derivatives in PBL patients. There is, however, a distinct requirement for novel prognostic tools to better delineate subgroups at risk for early relapse or refractory disease as well as late relapse. A comprehensive molecular characterization of a clinically annotated cohort of PBL patients is therefore urgently warranted.

**Supplementary Information:**

The online version contains supplementary material available at 10.1007/s00432-021-03580-z.

## Introduction

Plasmablastic lymphoma (PBL) is a rare preterminally differentiated B-cell malignancy with features resembling both high-grade B-cell lymphoma and plasma cell malignancies (Delecluse et al. 1997; Loghavi et al. 2015; Vega et al. 2005). PBL is predominantly encountered in the context of age-related immunosenescence, secondary to iatrogenic immunosuppression or in HIV-positive patients and the clinical course is commonly aggressive with dismal overall survival (OS) (Tchernonog et al. 2017).

Patients with PBL are insufficiently represented in clinical trials, treatment approaches are heterogeneous and translational research is challenging. Nevertheless, several retrospective studies identified a number of predictors of clinical outcome including age, performance status, HIV- and *MYC*-status (Miao et al. 2020; Tchernonog et al. 2017; Witte et al. 2020).

The International Prognostic Index (IPI), the most commonly used prognostic score in aggressive B-cell lymphoma, especially in diffuse large B-cell lymphoma (DLBCL), was first developed by Shipp et al*.*, more than 25 years ago, in the pre-rituximab era (International Non-Hodgkin's Lymphoma Prognostic Factors 1993). The analysis of more than 2000 patients with aggressive non-Hodgkin lymphoma treated with anthracycline-based regimens in the U.S., Canada and Europe led to the identification of five independent prognostic factors impacting survival: age (≤ 60 vs. > 60 years), stage (I/II vs. III/IV), number of extranodal (EN) sites (0–1 vs. ≥ 2), performance status (PS; 0–1 vs. ≥ 2) and serum lactate dehydrogenase (LDH; normal vs. elevated). Simultaneously, a scoring system for younger patients < 60 years of age was developed (age-adjusted IPI). In this subgroup, ECOG (Eastern Cooperative Oncology Group) Performance-Status, stage and LDH maintained their statistically significant impact on patient outcome, thereby stratifying patients into four risk groups (score 0–3) (International Non-Hodgkin's Lymphoma Prognostic Factors 1993). Nearly fifteen years later, Sehn et al. (2007) refined the initial four risk categories (low- (score 0–1), low– intermediate- (2), high–intermediate- (3) and high- risk (4–5)) to better reflect patient outcome in the rituximab era in a more precisely defined cohort of DLBCL (*n* = 365) patients according to the WHO classification of hematopoietic and lymphoid tumors, which resulted in three distinct categories (R-IPI: ‘Very good’ (score 0), ‘Good’ (1–2) and ‘Poor’ (3–5)). Shortly thereafter, a pooled analysis from several prospective clinical trials, drawing data from more than 1000 patients, demonstrated the IPIs continual prognostic capabilities despite the introduction of rituximab (Ziepert et al. 2010).

More recently, in an effort to identify a subpopulation of aggressive B-cell lymphoma patients with distinctly inferior outcome (5-year OS < 50%), despite immunochemotherapy, the IPI concept was further revised to incorporate both incremental age and LDH categories alongside the involvement of pre-defined extranodal sites (NCCN-IPI) (Zhou et al. 2014). Its proclaimed predictive superiority over the IPI/R-IPI has however been questioned, predominantly in the light of the advent of PET-based initial diagnosis and -guided therapy (El-Galaly et al. 2015). Additionally, recent reports suggest another potential refinement of the IPI through the integration of baseline beta2-microglobulin levels (GELTAMO-IPI) (Montalban et al. 2017).

However, unlike DLBCL for which there exist several validated prognostic indices, no such scoring system has been established for PBL, despite preliminary data from several retrospective investigations suggest informative applicability of the IPI in both HIV-positive and negative patients (Schommers et al. 2013; Tchernonog et al. 2017).

In the current study, we identified 78 PBL patients with available clinical data, for which in most cases (76/78) centralized hematopathological workup was available. We retrospectively evaluated clinicopathological characteristics and provide, to the best of our knowledge, the first comparison of prognostic indices ((aa)-IPI, R-IPI and NCCN-IPI) in this rare type of non-Hodgkin lymphoma.

## Methods

### Patients and treatment

We retrospectively reviewed our institutional hematopathological database to identify PBL patients whose biopsy specimen from initial diagnosis had been referred to the Reference center for Hematopathology University Hospital Schleswig Holstein Campus Lübeck and Hämatopathologie Lübeck for centralized histopathological panel evaluation between January 2000 and December 2018. Diagnosis was confirmed in a panel setting by three experienced hematopathologists (ACF, HM and HWB) in accordance with the current edition of the WHO classification of tumors of the hematopoietic and lymphoid tissues (Swerdlow et al. 2016). Antibodies and positivity cutoffs employed in the current study are summarized in Supplementary Table 1 and as described (Witte et al. 2020). Fluorescence in situ hybridization (FisH) for *MYC* was routinely performed, as described, wherever the biopsy was of sufficient size and quality (Gebauer et al. 2015).

In total, 78 consecutive patients with PBL (median age 63 years; range 26–91), were identified and assessed for clinicopathological baseline characteristics, including (age-adjusted in patients < 60 years)-IPI, R-IPI, NCCN-IPI, ARL-IPI and GELTAMO-IPI alongside therapy as well as outcome. These characteristics of the study group are briefly summarized in Table 1, Supplementary Tables 2, 3, and 4.

**Table 1:** Clinical characteristics of the study group

Characteristics	Plasmablastic lymphoma (*n* = 78)
Age (yrs.; median + range)	63 (26–91)
Sex	
Female	14 (78%)
Male	64 (82%)
B-symptoms	
Yes	47 (60%)
No	31 (40%)
Stage (Ann arbor)	
I/II	21 (27%)
III/IV	57 (73%)
ECOG PS	
0–1	33 (42%)
2–4	45 (58%)
LDH	
Normal	17 (22%)
Elevated ≤ 3x	36 (46%)
Elevated > 3x	25 (32%)
Extranodal sites	
0	12 (15%)
1–2	61 (78%)
> 2	5 (6%)
Bone marrow involvement	8 (10%)
HIV status	
Positive	30 (39%)
Negative	48 (62%)
IPI (International prognostic index)	
0–1 (low risk)	14 (18%)
2 (low-intermediate risk)	11 (14%)
3 (high-intermediate risk)	24 (31%)
4–5 (high risk)	29 (37%)
R-IPI (Revised-International prognostic index)	
0 (very good prognosis)	7 (9%)
1–2 (good prognosis)	18 (23%)
3–5 (poor prognosis)	53 (68%)
NCCN-IPI (National comprehensive cancer network-International prognostic index)	
0–1 (low risk)	3 (4%)
2–3 (low-intermediate risk)	17 (22%)
4–5 (high-intermediate risk)	28 (36%)
6–8 (high risk)	30 (39%)

*ECOG PS* Eastern Cooperative Oncology Group Performance Status, *LDH* lactate dehydrogenase, *yrs* years

This present study was approved by the ethics committee of the University of Lübeck (reference-no 18–311) and conducted in accordance with the declaration of Helsinki. Patients had given written informed consent regarding routine diagnostic and academic assessment of their biopsy specimen at the Reference center for Hematopathology and transfer of their clinical data. Most patients included in this analysis were previously investigated as part of a clinicopathological study, assessing the prognostic impact of *MYC*-translocation status (Witte et al. 2020).

### Statistics

Time to progression and overall survival (PFS, OS) were calculated from the date of initial diagnosis and censored at the date of last clinical contact. Survival (PFS and OS) according to prognostic risk groups was initially estimated by means of the Kaplan–Meier method and univariate log-rank test. Comparative analysis regarding performance of the prognostic indices was performed by employing the Harrel’s concordance index (c-index) (Heller and Mo 2016) and corrected Akaike’s Information Criterion (cAIC) (Akaike 1974; Sugiura 1978). While the c-index assess discriminatory power of a given statistical model (with higher values indicating superior predictive properties; ≤ 0.5 poor model, no better than predicting an outcome than random chance; 1 = perfect model, flawless prediction of outcome according to group allocation), the cAIC poses a means to quantify the predictive potential of statistical models upon direct comparison at low sample volumes (with lower values indicating better accuracy). A difference in cAIC values between 0 and 2 indicates the absence of significant differences in model fit while a difference between 2 and 10 suggests an increasing improvement in fit, a difference greater than 10 represents a substantial improvement in fit. All statistical investigations were conducted using GraphPad PRISM 6 and or R v4.0.2.

## Results

### Clinical characteristics of the study group

In total, 78 patients with PBL were retrospectively enrolled in this multi-center trial. Central histopathological review was available via the reference center for hematopathology Lübeck in 76/78 (97%) patients. Baseline clinical characteristics are provided in Table 1. Median age of the study group was 63 years with a pronounced male predominance (64/78; 82%). An underlying HIV infection was present in 30/78 patients (39%) and more than half of the study group presented with advanced stage disease (73% Ann Arbor III/IV) and reduced performance status (58% ECOG 2–4). The vast majority of patients presented with involvement of 1–2 extranodal sites with 5/78 (6%) patients presenting more than 2 extranodal manifestations and bone marrow infiltrates found in 8/78 (10%) patients. An elevation in LDH levels was found in 78% of patients (61/78) with 25 patients (32%) showing levels > three times the upper limit of norm.

While chemotherapeutic treatment approaches exhibited a substantial degree of variability, the dominant regimens of choice were CHOP or its derivates (55/78 patients 71%).

### International prognostic indices

Categorization according to pre-defined risk groups of all 78 patients was performed according to all three international prognostic indices. By means of the IPI 18% of patients were allocated to the low risk group, while 14% and 31% were low-intermediate or high-intermediate risk, respectively and 37% of patients were classified as high risk. In the updated R-IPI, initially proposed for aggressive B-cell lymphoma patients undergoing rituximab-containing therapy regimens, which was comparatively analyzed for internal validation and control purposes, as no improvement over the basic IPI was to be expected, we found 9% and 23% of patients to have a “very good” or “good” prognosis while 68% were allocated to the “poor” prognosis group.

According to the NCCN-IPI, only 3% were low risk and 22% and 36% were low-intermediate or high-intermediate risk, respectively. A high-risk score was found in 39%.

Agreement defined by weighted kappa (according to Cohen’s kappa coefficient) between IPI- and NCCN-IPI was moderate (weighted *k* = 0.57). Agreement in terms of risk category allocation was found in 54/78 (69%) patients, while 21/78 patients were grouped in adjacent risk categories. Reallocation according to the NCCN-IPI of patients initially stratified by IPI risk group is depicted in Supplementary Fig. 1. Inherent to the model design of IPI and R-IPI, due to identical individual risk factors, we observed a fixed pattern of reallocation as described (Ruppert et al. 2020; Sehn et al. 2007). Further, we calculated the AIDS-related lymphoma (ARL)-IPI in all HIV^+^ PBL patients for which sufficient clinical data was available (13/30) (Barta et al. 2014). Beyond the aaIPI (weighed two-fold), the ARL-IPI incorporates both involvement of extranodal sites and an HIV-score comprising viral load (copies per milliliter), the number of CD4 positive T-helper cells (number per microliter) and an individual prior history of AIDS (the latter factors both weighed one-fold). Fragmentary data prevented a meaningful statistical analysis in this population. Distribution of HIV^+^ PBL patients onto the ARL-IPI risk groups is delineated in Supplementary Table 2.

Similarly, the recent GELTAMO-IPI was only calculable in 31/78 patient due to missing baseline beta2-microglobulin levels in the majority of patients (Supplementary Table 4).

### Survival analysis

At a maximum follow-up of 175 months and a median follow-up of 14 months, we observed 54 deaths and a median overall survival of 17 months. The five-year survival rate was observed to be 21.98% (95% CI + 11.06/− 9.05%). Upon PFS analysis, we identified 58 events as well as a five-year PFS-rate of 21.14% (95% CI + 10.376/− 8.79%).

**Fig. 1:**
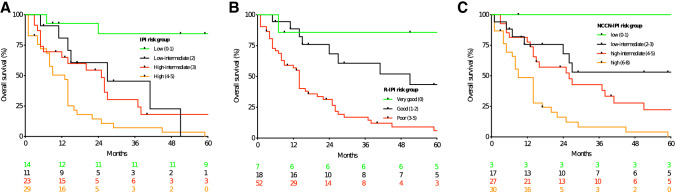
Overall survival according to the IPI (**a**), the R-IPI (**b**) and the NCCN-IPI (**c**)

**Fig. 2:**
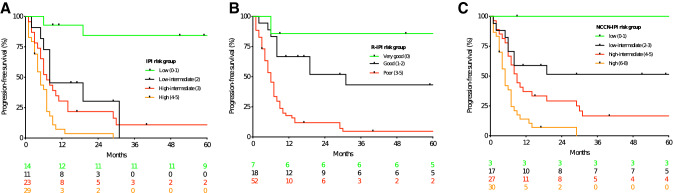
Progression-free survival according to the IPI (**a**), the R-IPI (**b**) and the NCCN-IPI (**c**)

As depicted in Fig. 1, all three scores resulted in clinically meaningful subgroups with significant differences in OS. Estimated five-year survival according to risk groups upon employing the IPI, R-IPI and the NCCN-IPI ranged from 0 to 84.4%, 6.0 to 85.7% and 0 to 100%, respectively. This signifies that the widest prognostic spectrum is covered by the NCCN-IPI. Similar observations were made regarding PFS analysis. These observations are summarized in Fig. 2.

Upon comparative analysis with both IPI and R-IPI, the NCCN-IPI best identifies both a small “lowest risk” group of patients with excellent outcome as well as a “highest risk” group of patients with pronounced early therapeutic failure.

### Comparative analysis of model fit and concordance

Model analysis according to both AIC as well as cAIC revealed ambiguous fit of the NCCN-IPI compared with both the R-IPI and the (aa)-IPI model. Differences were marginal, failing to reach the predefined level of significance in our minor cohort (cAIC differences < 2; range: 137.8–138.6).

Analysis of concordance index, however, revealed a clinically meaningful advantage in favor of the NCCN-IPI in the discrimination of patients with poor and favorable OS (signified by the highest, yet still insufficient c-index (NCCN-IPI: 0.564; IPI: 0.523; R-IPI: 0.5). Data on model fit and concordance are summarized for OS in Table 2 and results from an univariate analysis of statistical impact of individual risk factors included in the IPI, R-IPI and the NCCN-IPI on PBL patient overall survival in addition to their individual hazard ratio are depicted in Table 3. In addition, results from both uni- and multivariate analysis of statistical impact of individual risk factors included in the IPI, R-IPI and the NCCN-IPI on PBL patient OS in the context of their respective survival model (IPI/R-IPI and NCCN-IPI) are delineated in Table 4.

**Table 2:** Data on model fit and concordance are summarized for overall survival

Model	c-index (95% CI)	cAIC	Hazard ratio (95% CI)
IPI risk group	0.523	138.20	
Low (0–1)	(0.485–0.712)		Reference
Low-intermediate (2)			9.48 (2.23–40.33)
High-intermediate (3)			5.73 (2.25–14.59)
High (4–5)			7.86 (3.58–17.25)
R-IPI risk group	0.500	138.65	
Very good (0)	(0.409–0.640)		Reference
Good (1–2)			2.71 (0.66–11.09)
Poor (3–5)			3.84 (1.80–8.20)
NCCN-IPI risk group	0.564	137.84	
Low (0–1)	(0.498–0.723)		Reference
Low-intermediate (2–3)			3.33 (0.45–24.40)
High-intermediate (4–5)			3.44 (0.95–12.46)
High (6–8)			4.62 (1.59–13.43)

**Table 3:** Univariate analysis of statistical impact of individual risk factors included in the IPI, R-IPI and the NCCN-IPI on PBL patient overall survival

Risk factor	Hazard ratio (95% CI)	*p* value
Age (years) (IPI/R-IPI)		
≤ 60	Reference	0.002
> 60	2.356 (1.353–4.105)	
Age (years) (NCCN-IPI)		
≤ 40	Reference	0.0095
41–60	1.577 (0.662–3.759)	
61–75	2.310 (1.077–4.954)	
> 75	1.734 (0.681–4.418)	
Ann arbor		
I/II	Reference	0.005
III/IV	3.157 (1.411–7.061)	
ECOG		
≤ 1	Reference	< 0.0001
2–4	4.603 (2.477–8.554)	
LDH (ULN-ratio) (IPI/R-IPI)		
≤ 1	Reference	< 0.0001
> 1	6.107 (2.354–15.849)	
LDH (ULN-ratio) (NCCN-IPI)		
≤ 1	Reference	< 0.0001
1–3	3.663 (1.78–7.539)	
> 3	5.865 (2.521–13.65)	
Extranodal sites		
≤ 1	Reference	0.035
> 1	1.799 (1.042–3.106)	
Specific organ involvement*		
No	Reference	0.082
Yes	1.643 (0.938–2.875)	

*ECOG* Eastern Cooperative Oncology Group Performance Status, *LDH* lactate dehydrogenase

*Involvement of the bone marrow, central nervous system, liver or gastrointestinal system, or lung

**Table 4:** Uni- and multivariate analysis of statistical impact of individual risk factors included in the IPI, R-IPI and the NCCN-IPI on PBL patient overall survival in the context of their respective survival model (IPI/R-IPI and NCCN-IPI)

Risk factor	Univeriate analysis	Multivariate analysis	
	*p* value	Hazard ratio (95% CI)	*p* value
IPI/R-IPI			
Age	0.002	1.820 (0.994–3.334)	0.052
Stage I/II vs. III/IV	0.005	0.753 (0.276–2.054)	0.579
ECOG 0–1 vs. 2–4	< 0.0001	2.521 (1.217–5.220)	**0.013**
LDH < uln vs. > uln	< 0.0001	3.565 (1.036–12.264)	**0.044**
Extranodal sites 0–1 vs. > 1	0.035	1.621 (0.910–2.888)	0.101
NCCN-IPI			
Age (groups 1–4)	0.0095	1.260 (0.905–1.754)	0.171
Stage I/II vs. III/IV	0.005	1.035 (0.390–2.745)	0.944
ECOG 0–1 vs. 2–4	< 0.0001	3.049 (1.383–6.724)	**0.006**
LDH (groups 1–3)	< 0.0001	1.677 (0.982–2.863)	0.058
Specific extranodal sites*	0.082	2.156 (1.177–3.949)	**0.013**

Bold values indicate statistically significance *p* ≤ 0.05

*ECOG* Eastern Cooperative Oncology Group Performance Status, *LDH* lactate dehydrogenase

*Involvement of the bone marrow, central nervous system, liver or gastrointestinal system, or lung

In summary, our data underline the NCCN-IPI’s moderate clinical utility whilst prompting its recommendation for preferential use in clinical practice for the time being while concomitantly characterizing the IPI as a viable option in the context of retrospective studies, facing fragmentary datasets.

## Discussion

In the current study, we confirm that the original IPI clearly separates four prognostically divergent risk groups among one of the largest cohorts of PBL patients published to date regardless of HIV-status. Expectedly, no meaningful prognostic insight was gained through the application of the R-IPI (International Non-Hodgkin's Lymphoma Prognostic Factors 1993; Sehn et al. 2007). The NCCN-IPI, however, displayed superior, yet still insufficient concordance with comparable model fitting in our study group. In addition, the NCCN-IPI appears to cover the widest prognostic spectrum, allowing for a clearer distinction of patients with both most favorable and poorest OS. Similar observations were made regarding PFS analysis. Unlike recent observations in DLBCL, the NCCN-IPI reliably identified patients with both five-year OS- and PFS-rates below 20% (Ruppert et al. 2020; Zhou et al. 2014). Nevertheless, both concordance and fitting measures illustrate but moderate clinical utility of all three established indices, leaving significant room for improvement.

The more detailed recognition of patient age, included in the NCCN-IPI appears to be biased, regarding the underlying HIV-status (see Table 3). Its integration into clinical prognostication models appears to be less mandatory in PBL compared with recent data taken from an integrative analysis of seven prospective trials in DLBCL (Ruppert et al. 2020). None of the established scores, however, reflect the bilateral epidemiological spectrum of PBL with elderly patients developing PBL as an indicator of senile immunosenescence or immunosuppressive treatment in the context of organ transplant and significantly younger PBL patients harboring an underlying acquired immune deficiency syndrome (AIDS).

Upon biphasic multivariate analysis of the individual factors of all three scoring systems (IPI and R-IPI followed by the more specific incremental variables required to calculate the NCCN-IPI) we found that the effect of stage I/II vs. III/IV was non-independent, plausibly attributable to the inherent extranodal nature of PBL. Moreover, we found, that recognition of the specific subset of involved sites included in the NCCN-IPI was superior to the cut-off of 0–1 vs. > 1 extranodal sites in the (R-) IPI, whereas statistical significance and independence was lost upon incremental evaluation of LDH levels, calling into question this particular refinement of the NCCN-IPI in PBL prognostication.

In a recent meta-analysis evaluating the prognostic capabilities of IPI, R-IPI and NCCN-IPI in R-CHOP treated DLBCL patients, four out of the initial 14 trails extracted from the SEAL database had to be excluded due to missing data regarding extranodal sites of involvement (Ruppert et al. 2020). This suggests that despite all prognostic indices evaluated in this study being based on readily available clinical data, fragmentary documentation, especially in patients treated prior to the introduction or widespread implementation of the NCCN-IPI may pose an issue.

More so, established scoring systems fail to identify signs of refractory disease as well as PBL patients at risk of late relapse with sufficient statistical significance. In DLBCL, similar requirements were recently addressed successfully through the integrative analysis of clinical, molecular and cytogenetic data (Chapuy et al. 2018; Schmitz et al. 2018). Beyond recent advances regarding the prognostic role of *MYC* in PBL, similar data are warranted (Witte et al. 2020).

The shortcomings, inherent to such a retrospective study design, as well as the therapeutic variability, common to PBL treatment approaches, are acknowledged. This unfortunately prevented a meaningful analysis regarding the ARL-IPI in the HIV^+^ subgroup of our cohort. This model was however conceptualized in CD20^+^ lymphoma patients in the rituximab-era and its contributing, lymphoma-related risk factors resemble the aa-IPI which we revealed to show inferior concordance, compared to the NCCN-IPI. In prospectively assessed cohort of HIV^+^ PBL patients it would appear preferable to investigate the addition of the ARL-IPI HIV score into the NCCN-IPI. Moreover, it would be of interest to investigate the predictive properties of the GELTAMO-IPI in a cohort with sufficient information on baseline beta-2-microglobulin levels (Montalban et al. 2017).

In summary, our observations support the use of the IPI and more so its enhanced derivative NCCN-IPI in PBL patients. There is, however, a distinct requirement for novel prognostic tools to better delineate subgroups at risk for early relapse or refractory disease as well as late relapse. A comprehensive molecular characterization of a clinically annotated cohort of PBL patients is therefore urgently warranted to identify additional risk factors, advance baseline prognostication and potentially treatment guidance.

## Supplementary Information

Below is the link to the electronic supplementary material.

Supplementary file1 (DOCX 49 KB)

## Data Availability

No individual participant data will be available automatically. No other documents will be available. Upon request, underlying dataset would be provided for individual participant data meta-analysis.
