# Supplementary material for: Performance of international prognostic indices in plasmablastic lymphoma: a comparative evaluation
Source: J Cancer Res Clin Oncol. 2021 Mar 3;147(10):3043–50. doi: 10.1007/s00432-021-03580-z (PMC8397630; doi:10.1007/s00432-021-03580-z)
Supplement: Supplementary file 1 — Supplementary file1 (DOCX 49 KB) [file 432_2021_3580_MOESM1_ESM.docx]

**Supplementary Table 1.** Antibodies used

| **Antibody** | **Supplier** | **Clone** | **Positivity cutoff** |
| --- | --- | --- | --- |
| Bcl2  | Lab Vision | 100/D5 | 30% |
| Bcl6 | Dako | BG-B6p | 30% |
| CD10 | Menarini | 56C6 | 30% |
| CD20 | Dako | L26 | - |
| CD30 | Dako | BerH2 | 10% |
| CD38 | Leica Biosystems | SPC32 | - |
| CD138 | Leica Biosystems | MI15 | - |
| CD56 | Leica Biosystems | CD564 | 10% |
| Kappa | Leica Biosystems | CH15 | - |
| Lambda | Leica Biosystems | SHL53 | - |
| MUM-1 (Irf4) | Dako | Mum 1P | 30% |
| Ki-67 | Dako | Mib-1 | - |
|  | | | |

**Supplementary Table 2.** Distribution of HIV^+^ PBL patients onto the ARL-IPI risk groups and individual risk factors.

| **Characteristics** | **HIV-positive PBL patients n =30/78** |
| --- | --- |
| **Fully available datasets** | n =13 (43%) |
| **Age (yrs; median, range)** | 42 (29 - 64) |
| **Sex** | |
| Female | 2 (15%) |
| Male | 11 (85%) |
| **aaIPI** | |
| 0 | 3 (23%) |
| 1 | 1 (8%) |
| 2 | 2 (15%) |
| 3 | 7 (54%) |
| **Extranodal Sites** | |
| 0 | 2 (15%) |
| 1 | 6 (46%) |
| 2 | 4 (31%) |
| ≥ 3 | 1 (8%) |
| **HIV-Score** | |
| Viral load (copies/ml; median, range) | 30 900 (300 - 3 500 000) |
| Viral load Score (median, range) | 2 (0 -2) |
| CD4 count (cells/*u*l; median, range) | 150 (24 - 390) |
| CD4 Score (median; range) | 2 (1 - 3) |
| History of AIDS | 9 (70%) |
| Calculation (median; range) | 4 (1 - 6) |
| **ARL-IPI (median; range)** | 10 (5 - 13) |
| Low (0 - 6) | 3 (23%) |
| Intermediate (7 - 10) | 5 (39%) |
| High (11 - 15) | 5 (39%) |
| aaIPI, age-adjusted International prognostic Index; AIDS, acquired immune deficiency syndrome; HIV, human immunodeficiency virus; PBL, plasmablastic lymphoma; yrs, years.  HIV-score (0-6): composite score base-line CD4 count (cells/uL): <50 =3, 50-199=2, 200-499=1, ≥500=0; HIV viral load (copies/mL): <400=0; 400-9,999=1, ≥10,000=2; and prior history of AIDS=1. ‡ARL-IPI=([aaIPI]x2)+[ENS]+[HIV-score]=0-15. | |

**Supplementary Table 3.** Distribution of the aa-IPI in the respective study population of PBL patients and representation of individual risk factors.

| **Characteristics** | **aaIPI (n = 37)** |
| --- | --- |
| **Age (yrs; median, range)** | 44 (26-60) |
| **Sex** | |
| Female | 5 (14%) |
| Male | 32 (87%) |
| **ECOG-PS** | |
| 0-1 | 23 (62%) |
| 2-4 | 14 (38%) |
| **Ann-Arbor stage** | |
| I/II | 13 (35%) |
| III/IV | 24 (65%) |
| **LDH** | |
| Normal (≤240 U/l) | 10 (27%) |
| Elevated (>240 U/l) | 27 (73%) |
| **aa-IPI (median; range)** |  |
| Low (0) | 9 (24%) |
| Low-intermediate (1) | 3 (8%) |
| High-intermediate (2) | 13 (31%) |
| High (3) | 12 (32%) |
| aaIPI, age-adjusted International prognostic Index; ECOG-PS, Eastern Cooperative Oncology Group Performance Status; LDH, lactatedehydrogenase; PBL, plasmablastic lymphoma; yrs, years. | |

**Supplementary Table 4.** Distribution of the GELTAMO-IPI in the respective study population of PBL patients and representation of individual risk factors.

| **Characteristics** | **GELTAMO-IPI(n = 32)** |
| --- | --- |
| **Age (yrs; median, range)** | 63 (32-86) |
| <65 | 17 (53%) |
| 65 - 79 | 10 (31%) |
| ≥80 | 5 (16%) |
| **Sex** | |
| Female | 8 (25%) |
| Male | 24 (75%) |
| **ECOG-PS** | |
| 0-1 | 11 (34%) |
| 2-4 | 21 (66%) |
| **Ann-Arbor stage** | |
| I/II | 8 (25%) |
| III/IV | 24 (75%) |
| **LDH** | |
| Normal (≤240 U/l) | 6 (19%) |
| Elevated (>240 U/l) | 26 (81%) |
| **Beta-2-microglobulin, normalized ratio** | |
| ≤1 | 6 (19%) |
| >1 | 26 (81%) |
| **GELTAMO-IPI (median; range)** |  |
| Low (0) | - |
| Low-intermediate (1-3) | 10 (31%) |
| High-intermediate (4) | 7 (22%) |
| High (≥5) | 15 (47%) |
| ECOG-PS, Eastern Cooperative Oncology Group Performance Status; GELTAMO, Grupo Espanol de Linfomas y Transplantes de Médula Ósea; IPI, International prognostic Index; LDH, lactatedehydrogenase; PBL, plasmablastic lymphoma; yrs, years. | |

**Supplementary Figure 1:** Reallocation according to NCCN-IPI risk groups of patients primarily stratified according to the IPI.
